# 3 Jahre Cannabis als Medizin – Zwischenergebnisse der Cannabisbegleiterhebung

**DOI:** 10.1007/s00103-021-03285-1

**Published:** 2021-02-09

**Authors:** Gabriele Schmidt-Wolf, Peter Cremer-Schaeffer

**Affiliations:** Bundesinstitut für Arzneimittel und Medizinprodukte, Bundesopiumstelle, Kurt-Georg-Kiesinger-Allee 3, 53175 Bonn, Deutschland

**Keywords:** Cannabisarzneimittel, Verordnung, Begleiterhebung, Indikationen, Nebenwirkungen, Medical cannabis, Prescription, Interim evaluation, Indications, Side effects

## Abstract

In Deutschland sind Ärztinnen und Ärzte, die Cannabisarzneimittel zulasten der gesetzlichen Krankenversicherung verschreiben, verpflichtet, an einer bis zum 31.03.2022 laufenden nichtinterventionellen Begleiterhebung zur Anwendung dieser Cannabisarzneimittel teilzunehmen.

Zum Zeitpunkt der Zwischenauswertung am 11.05.2020 lagen 10.010 vollständige Datensätze vor. Am häufigsten wurden Schmerzen (73 %) als primär therapierte Symptomatik genannt, gefolgt von Spastik (10 %) sowie Anorexie/Wasting (6 %). Verordnet wurden in 65 % der Fälle Dronabinol (z. B. als Rezeptur oder Marinol® [AbbVie, IL, USA]), in 18 % Cannabisblüten, in 13 % Sativex® (GW Pharma, Cambridge, UK), in 0,3 % Nabilon (z. B. Canemes® [AOP Orphan Pharmaceuticals AG, Wien, Österreich]) und in 4 % (mit zunehmender Tendenz) Cannabisextrakt. Die Fallzahl von 6485 Fällen erlaubt bei Dronabinol bereits die Auswertung von Subgruppen hinsichtlich der therapeutischen Wirksamkeit. Für Cannabis typische Nebenwirkungen, wie Müdigkeit, Schwindel, Schläfrigkeit, Übelkeit und Mundtrockenheit, traten bei der Verwendung aller Cannabisarzneimittel auf und sind bereits aus den Fachinformationen der cannabisbasierten Fertigarzneimittel bekannt. Potenziell schwerwiegende Nebenwirkungen wie Depression, Suizidgedanken, Wahnvorstellungen, Halluzinationen, Dissoziation und Sinnestäuschungen sind in jeweils mehr als einem von 1000 Fällen gemeldet worden.

Mit Cannabisblüten behandelte Personen sind deutlich jünger und weisen einen erheblich höheren Männeranteil auf. Sie werden häufiger hausärztlich behandelt (Allgemeinmedizin, innere Medizin), weichen häufiger von den üblicherweise behandelten Diagnosen (Schmerz, Spastik und Anorexie/Wasting) ab und verfügen über mehr Vorerfahrungen mit Cannabis. Das in der Begleiterhebung bestehende Underreporting betrifft diese Fallgruppe am stärksten.

## Abstract (en)

In Germany, physicians who prescribe medical cannabis flowers or cannabis-based medicines (narcotic prescription) at the expense of the statutory health insurance are obliged to take part in a noninterventional accompanying survey that runs until 31 March 2022.

At the time of this interim evaluation, 11 May 2020, there were 10,010 complete datasets collected. The most frequently treated symptoms were pain (73%), followed by spasticity (10%), and anorexia/wasting (6%). Dronabinol (i.e., prescription drug or Marinol®) was most frequently (65%) prescribed, followed by cannabis flowers (18%), Sativex® (13%), cannabis extract (4%, with increasing frequency), and nabilone (0.3%). The 6485 cases treated with dronabinol already allows a subgroup evaluation concerning efficacy. The typical cannabis side effects of tiredness, dizziness, dry mouth, and nausea occur with all cannabis medicines, and correspond to those already known from the product information of the cannabis-based medicinal products authorized under the pharmaceutical law. The potentially serious adverse effects of depression, suicidal ideation, delusions, hallucinations, dissociation, and misperceptions were each reported with a frequency higher than 0.1%. There were remarkable differences between patients treated with cannabis flowers and those with other cannabis medicines.

Patients treated with cannabis flowers are significantly younger and predominantly male. They are treated more often by general practitioners and internists, their diagnosis differs more often from the typical diagnoses (pain, spasticity, anorexia/wasting), and they have more prior experience with cannabis. The underreporting in the accompanying survey is mainly in this patient group.

## Hintergrund

Ärztinnen und Ärzte, die Cannabisarzneimittel zulasten der gesetzlichen Krankenversicherung verschreiben, sind seit dem 10.03.2017 verpflichtet, an einer bis zum 31.03.2022 laufenden nichtinterventionellen Begleiterhebung zur Anwendung dieser Arzneimittel teilzunehmen. Die Erhebung wird vom Bundesinstitut für Arzneimittel und Medizinprodukte (BfArM) durchgeführt. Zur Historie und den gesetzlichen Grundlagen verweisen wir auf die erste Zwischenauswertung der Begleiterhebung im Mai 2019 [1]. Zum Stichtag der Auswertung am 11.05.2020 lagen mehr als 10.000 Datensätze vor, sodass eine Aktualisierung der Zwischenergebnisse und eine differenziertere Betrachtung möglich sind.

Die Ergebnisse der Begleiterhebung werden unter anderem Grundlage für den Gemeinsamen Bundesausschuss (G-BA) sein, um die zukünftige Übernahme der Behandlungskosten im Rahmen einer Therapie mit Cannabisarzneimitteln nach dem Sozialgesetzbuch (SGB) V zu regeln.

Ziele der Erhebung sind das Monitoring von Nebenwirkungen und Verträglichkeit, eine Abschätzung der Häufigkeitsverteilung von Indikationen, die eine Behandlung mit Cannabisarzneimitteln begründen, und eine orientierende Beurteilung der Effizienz als Grundlage für die Planung klinischer Studien.

Mit dieser Zwischenauswertung werden die Erkenntnisse aus der ersten Zwischenauswertung, die etwas mehr als 4000 Datensätze umfasste, überprüft und erweitert. Besondere Aufmerksamkeit wird dabei auf Nebenwirkungen und die Therapie mit Cannabisblüten gerichtet, die inzwischen mit einer relevanten Fallzahl erfasst ist.

## Datengrundlage und Methoden

Patientenbezogene Daten werden seit April 2017 über ein Onlineportal an das BfArM übermittelt. Die Daten sind im Rahmen der Begleiterhebung je Patientin oder Patient und verordnetem Cannabisarzneimittel höchstens 2‑mal im Verlauf der Therapie zu erheben. Die erste Erhebung erfolgt nach einer Therapiedauer von einem Jahr oder, wenn die Therapie mit dem gewählten Cannabisarzneimittel vor Ablauf eines Jahres abgebrochen wird, direkt nach Abbruch der Therapie. Für Versicherte, die sich nach dem 31.12.2021 in Therapie befinden, muss bis spätestens zum 31.03.2022 ein weiterer Erhebungsbogen ausgefüllt werden, unabhängig davon, ob bereits zuvor zu den gleichen Versicherten Daten übermittelt wurden [2]. Die Teilnahme an der Begleiterhebung ist verpflichtend.

Es werden anonymisierte Daten von Patienten/Patientinnen erfasst, die über eine Zusage der Kostenübernahme für die Therapie mit Cannabisarzneimitteln durch die zuständige gesetzliche Krankenkasse verfügen. Die Datenerfassung erfolgt mit der Onlineumfrageapplikation LimeSurvey, die Auswertung mit der Statistik- und Analysesoftware SPSS® (IBM®) und die Tabellenkalkulation mit Microsoft Excel. Die Auswertung erfolgte zunächst deskriptiv. Cannabisblüten, Cannabisextrakte, Dronabinol, Nabilon und das Fertigarzneimittel Sativex® werden getrennt voneinander ausgewertet. Während es sich bei Dronabinol um reines Tetrahydrocannabinol (THC) handelt und Nabilon ein dem THC ähnliches synthetisches Cannabinoid darstellt, sind die Blüten und Extrakte sowie Sativex® als Stoffgemische zu bezeichnen. Cannabisblüten werden über den THC- und Cannabidiol-(CBD-)Gehalt definiert, während die Extrakte auf bestimmte Gehalte der Hauptwirkstoffe THC und CBD eingestellt sind.

Der Markt hinsichtlich der Cannabisblüten und verschiedener -extrakte ist dynamisch. Inzwischen werden einige Dutzend Sorten mit unterschiedlicher Wirkstoffzusammensetzung von verschiedenen Herstellern auf den Markt gebracht und z. T. auch wieder vom Markt genommen. Eine Auswertung zum Gebrauch einzelner Blütensorten und Extrakte ist bei der vorliegenden Fallzahl noch nicht möglich.

Seit April 2018, ein Jahr nach Beginn der Datenerfassung, gehen Daten von Patienten/Patientinnen zur Auswertung ein, die mehr als 12 Monate mit dem gleichen Cannabisarzneimittel behandelt wurden. Erste vorläufige Zwischenergebnisse wurden bereits veröffentlicht [1, 3].

Ausgewertet wurden, neben den demografischen Daten zu Alter und Geschlecht, die Haupt- und Nebendiagnosen, die vor der Anwendung von Cannabisarzneimitteln angewendeten Therapieformen, Begleittherapien, die Nebenwirkungen und die primär therapierte Symptomatik sowie der Therapieerfolg bzw. die Gründe für einen Therapieabbruch. Obligat ist die Angabe mindestens einer Diagnose nach der International Classification of Diseases-Revision 10- German Modification (ICD-10-GM), die zur Verordnung von Cannabisarzneimitteln geführt hat. Bis zu 3 Hauptdiagnosen und optional bis zu 3 Nebendiagnosen, die zusätzlich für die Beurteilung der Therapie mit Cannabisarzneimitteln relevant sind, können eingetragen werden.

## Ergebnisse

Zum Zeitpunkt der Zwischenauswertung am 11.05.2020 waren insgesamt 10.010 vollständige Datensätze eingegangen. In 6485 Fällen (65 %) wurde Dronabinol (z. B. als Rezeptur oder Marinol®), in 1818 Fällen (18 %) Cannabisblüten, in 1309 Fällen (13 %) Sativex®, in 29 Fällen (0,3 %) Nabilon (z. B. Canemes®) und in 369 Fällen (4 %) Cannabisextrakt verordnet.

### Verordnung

**Figure 1:**
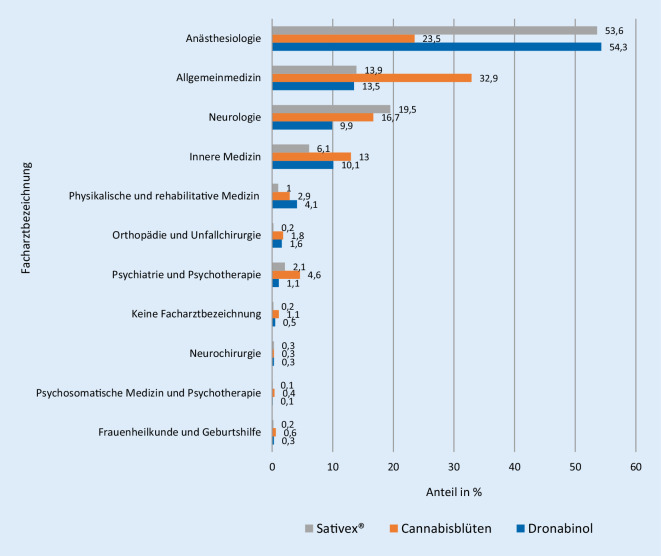

Nach Auswertung der 10.010 Datensätze erfolgte die Verordnung von Cannabisarzneimitteln hauptsächlich in den Fachbereichen Anästhesiologie und Allgemeinmedizin (49 % bzw. 17 %). Internisten/Internistinnen hatten einen Anteil von 10 % an den Verordnungen, Neurologen/Neurologinnen 12 %. Die Anteile der Facharztbezeichnungen bei Verschreibung von Dronabinol, Cannabisblüten bzw. Sativex® werden in Abb. 1 vergleichend dargestellt.

56 % der verordnenden Ärzte/Ärztinnen führten die Zusatzbezeichnung *spezielle Schmerztherapie*, 39 % waren in der *Palliativmedizin* weitergebildet. In der Anästhesie wurde in 72 % der Fälle Dronabinol verordnet (Sativex®: 14 %, Cannabisblüten: 9 %, Cannabisextrakt: 5 %), in der Neurologie wurde relativ häufig Sativex® eingesetzt (21 % vs. Dronabinol: 52 %, Cannabisblüten: 25 %, Cannabisextrakt: 2 %) und in der Allgemeinmedizin verstärkt Cannabisblüten (35 % vs. Dronabinol: 52 %, Sativex®: 11 %, Cannabisextrakt: 2 %) zu therapeutischen Zwecken.

Cannabisarzneimittel wurden Männern und Frauen insgesamt gleich häufig verschrieben. Der Anteil männlicher Patienten ist bei der Verschreibung von Cannabisblüten (68 %) deutlich höher als bei Dronabinol (42 %), Sativex® (46 %) und Cannabisextrakt (46 %). In 14 Fällen wurde das Geschlecht weder als männlich noch als weiblich angegeben. Bezogen auf alle 10.010 Datensätze waren Patienten/Patientinnen, denen Cannabisblüten verschrieben wurden, mit (im Median) 46 Jahren deutlich jünger als solche, die andere Cannabisarzneimittel erhalten haben (Dronabinol 60 Jahre, Sativex® 57 Jahre, Cannabisextrakt 57 Jahre).

### Therapieabbrüche

Die Therapie wurde bei 3499 Personen (35 %) innerhalb eines Jahres abgebrochen (bei Hauptindikation Schmerz: 34 %, Spastik: 25 %, Anorexie/Wasting: 57 %). Der Altersdurchschnitt bei den Therapieabbrechern (Median 63 Jahre) lag deutlich höher als der bei Personen, die mehr als ein Jahr mit einem Cannabisarzneimittel therapiert wurden (Median 54 Jahre). Die Therapie wurde bei Frauen häufiger abgebrochen als bei Männern (55 % vs. 45 %). Wurde die Therapie mit Cannabisarzneimitteln abgebrochen, erfolgte dies in 25 % der Fälle aufgrund von Nebenwirkungen, in 21 %, weil der Patient/die Patientin verstarb, in 3 %, weil keine weitere Therapienotwendigkeit für Cannabisarzneimittel bestand, in 0,3 % wegen Wechselwirkungen und in 12 % aus anderen Gründen. Häufigster Abbruchgrund war jedoch mit 39 % die nicht ausreichende Wirkung (Schmerz 45 %, Spastik 40 %, Anorexie/Wasting 16,7 %).

### Behandlungsindikationen

73 % aller Verordnungen von Cannabisarzneimitteln wurden zur Behandlung von Schmerzen, 10,3 % zur Besserung einer Spastik, 6,4 % zur Therapie von Anorexie/Wasting und 10,3 % zur Behandlung einer anderen Symptomatik angewendet. Eine Übersicht zu den relevanten Hauptdiagnosen, die eine Behandlung mit Cannabisarzneimitteln begründen, gibt Tab. 1.

**Table 1:** 

Erkrankung bzw. Symptomatik	Fallzahl^a,b^, (*n* = 10.010)	Relativer Anteil in %
Schmerz	7312	73
Neubildung	1831	18
Spastik	1028	10
Anorexie/Wasting	641	6
Multiple Sklerose	607	6
Übelkeit/Erbrechen	511	5
Depression	279	3
Migräne	207	2
ADHS	113	1
Appetitmangel/Inappetenz	119	1
Darmkrankheit, entzündlich, nichtinfektiös	121	1
Epilepsie	104	1
Ticstörung inkl. Tourette-Syndrom	82	<1
Restless-legs-Syndrom	90	<1
Insomnie/Schlafstörung	86	<1
Clusterkopfschmerz	59	<1

^a^Grundlage für die Berechnung sind bei ICD-10-Codes die bis zu 3 Hauptdiagnosen

^b^Mehrfachnennungen sind möglich, zum Beispiel werden Patientinnen/Patienten mit den Diagnosen Migräne oder Clusterkopfschmerz in der Regel auch unter der Diagnose Schmerz erfasst

In 178 Fällen (1,8 %) wurde zusätzlich der ICD-10-Code Z51.5 (Palliativbehandlung) eingegeben, bei Behandlung mit Dronabinol in 142 Fällen (2,2 %), mit Cannabisblüten in 16 Fällen (0,9 %) und mit Sativex® in 13 Fällen (1,0 %).

**Figure 2:**
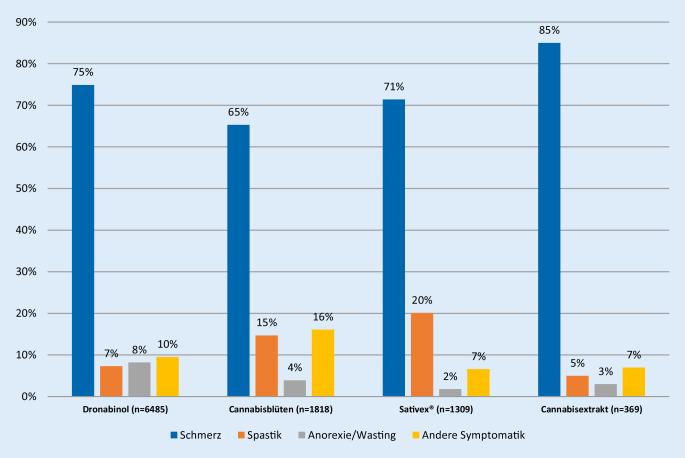

Dronabinol wurde in 75 % der Fälle primär zur Behandlung von Schmerzen verordnet, Sativex® zu 71 %, Cannabisblüten zu 65 % und Cannabisextrakt zu 85 % (Abb. 2). Dronabinol diente in 8 % der Fälle zur Behandlung von Anorexie/Wasting, Sativex® in 2 %, Cannabisblüten in 4 % und Cannabisextrakt in 3 %. Cannabisblüten wurden mit 16 % häufiger zur Behandlung anderer Symptome als Schmerz, Spastik und Anorexie eingesetzt (Dronabinol: 10 %, Sativex®: 7 %, Cannabisextrakt: 7 %). Sativex® wurde, in Anlehnung an die Indikation bei multipler Sklerose, mit 20 % relativ häufig zur Behandlung einer Spastik verschrieben (Cannabisblüten: 15 %, Dronabinol: 7 %, Extrakte: 5 %; Abb. 2).

#### Indikationen bei Krebserkrankungen

Insgesamt lag bei 1831 Patientinnen und Patienten (18 %) eine bösartige Neubildung vor. Bei 989 dieser Personen wurde die Therapie vor Ablauf eines Jahres abgebrochen, davon sind 562 verstorben. In 160 Fällen (8,8 %) wurde zusätzlich der ICD-10-Code Z51.5 (Palliativbehandlung) eingegeben. Der Median für das Alter der Krebserkrankten betrug 63 Jahre, der Anteil der Männer 48 %.

Weitaus am häufigsten (80 %) wurde Dronabinol verschrieben, gefolgt von Cannabisblüten mit 11 % und Sativex® mit 5 %. Bei 49 % der Behandelten wurde primär die Symptomatik Schmerz therapiert, bei 27 % Anorexie/Wasting, bei 2 % Spastik und bei 12 % Übelkeit/Erbrechen. Bei 10 % der Krebspatienten wurden eher unspezifische Symptome, wie z. B. Schlafstörungen, Unruhe, Anspannung, Fatigue und Appetitmangel, als primäres Symptom behandelt.

In allen 4 ausgewerteten Symptomkategorien wurde der Therapieerfolg am häufigsten als *moderat verbessert* eingeschätzt. Bei den 227 Fällen mit Übelkeit/Erbrechen an erster Stelle der primär behandelten Symptomatik wurde in 34 % der Fälle eine deutliche Verbesserung berichtet.

Der Anteil der Patienten mit Tumorerkrankung an der Gesamtzahl der Schmerzpatienten in der Begleiterhebung betrug 12,4 % (905 von 7312 Fällen), bei der primär behandelten Symptomatik Spastik 3,7 % (38 von 1028 Fällen), bei der primär behandelten Symptomatik Anorexie/Wasting 76,6 % (491 von 641 Fällen) und bei der Behandlung von Übelkeit/Erbrechen als primäres Symptom 85 % (227 von 267 Fällen). Wenn Übelkeit/Erbrechen auch in Kombination mit anderen Symptomen behandelt wurde, betrug der Anteil der Patienten mit Tumorerkrankung an der Gesamtzahl 79 % (403 von 511).

### Erkrankungs- und Behandlungsdauer, Therapieverfahren vor der Behandlung

Der Median für die Dauer der Erkrankung, die eine Behandlung mit Cannabisarzneimitteln erforderlich machte, betrug 96 Monate (Dronabinol: 84 Monate, Cannabisblüten: 120 Monate, Sativex®: 108 Monate). Der Median für die Behandlungsdauer betrug 10 Monate (Dronabinol: 8 Monate, Cannabisblüten: 12 Monate, Sativex®: 8 Monate, Cannabisextrakte: 12 Monate). In 17 % der Fälle wurde maximal ein Monat mit Cannabisarzneimitteln therapiert (Dronabinol: 19 %, Cannabisblüten: 12 %, Sativex®: 18 %, Cannabisextrakte: 13 %).

Bezüglich der Therapieverfahren, die vor Beginn einer Behandlung mit Cannabisarzneimitteln durchgeführt wurden (z. B. Opioidtherapie, physikalische Therapie, operative Verfahren), lassen sich zwischen den einzelnen Cannabisarzneimitteln keine relevanten Unterschiede feststellen.

### Nebenwirkungen

**Figure 3:**
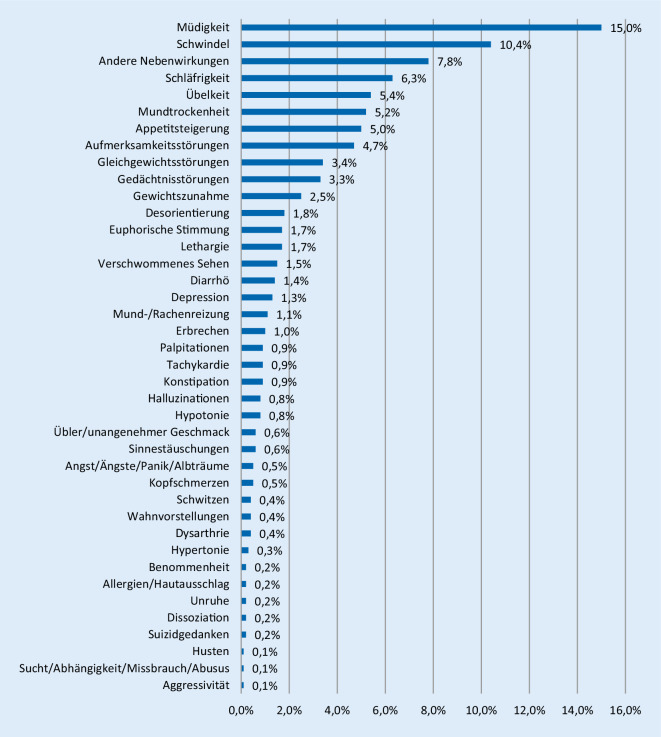

Für Cannabis typische Nebenwirkungen, wie Müdigkeit, Schwindel, Schläfrigkeit, Mundtrockenheit und Übelkeit, traten bei der Verwendung aller Cannabisarzneimittel häufig, z. T. sehr häufig auf (Abb. 3). Auch die weiteren für alle Cannabisarzneimittel gemeldeten Nebenwirkungen sind aus den Fachinformationen zu den Fertigarzneimitteln Sativex® [4] und Canemes® [5] oder auch des u. a. in den USA zugelassenen Marinol® (Dronabinol; [6]) bekannt. Die potenziell schwerwiegenden Nebenwirkungen Suizidgedanken (0,2 %, d. h. 1 Fall von 500, gelegentlich), Depression (1,3 %, d. h. > 1 Fall von 100, häufig), Halluzinationen (0,8 %, d. h. < 1 Fall von 100, gelegentlich), Dissoziation (0,2 %, d. h. 1 Fall von 500, gelegentlich) und Wahnvorstellungen (0,4 %, d. h. 1 Fall von 250, gelegentlich) wurden in der Begleiterhebung nicht selten gemeldet. Kopfschmerz (50 Fälle), Unruhe (18 Fälle), Aggression (11 Fälle), Benommenheit (24 Fälle), Konzentrationsstörungen (10 Fälle), Angst (27 Fälle), Panik (15 Fälle), Albträume (10 Fälle), Allergien (16 Fälle), Husten (14 Fälle) traten jeweils gelegentlich auf, kardiale Symptome (9 Fälle) sowie Sucht/Abhängigkeit/Missbrauch/Abusus (6 Fälle) wurden im Freitext als seltene Nebenwirkungen berichtet.

Bei Sativex® wurden vergleichsweise häufig Mundtrockenheit und Übelkeit (übler/unangenehmer Geschmack 57 Fälle) sowie durch die Anwendung als Mundspray bedingte Unverträglichkeiten an den Schleimhäuten (Mund‑/Rachenreizung 105 Fälle) berichtet.

Die Nebenwirkungsprofile von Dronabinol (als reines THC) und den weiteren (mit Ausnahme von Nabilon) sämtlich CBD-haltigen Cannabisarzneimitteln unterschieden sich nicht wesentlich (Tab. 2).

**Table 2:** 

	Dronabinol (*n* = 6485) (in %)	CBD-haltige CAM (*n* = 3496) (in %)
Müdigkeit	14,8	15,4
Schwindel	11,1	9,0
Andere Nebenwirkungen	6,9	11,0
Schläfrigkeit	6,6	5,6
Übelkeit	5,5	5,2
Appetitsteigerung	4,9	5,2
Aufmerksamkeitsstörungen	4,6	4,8
Mundtrockenheit	3,9	7,8
Gleichgewichtsstörungen	3,6	2,9
Gedächtnisstörungen	3,6	2,8
Gewichtszunahme	2,7	2,2
Desorientierung	2,0	1,6
Verschwommenes Sehen	1,6	1,4
Lethargie	1,6	1,7
Diarrhö	1,4	1,2
Euphorische Stimmung	1,3	2,5
Depression	1,1	1,6
Erbrechen	1,0	1,0
Konstipation	0,9	0,9
Tachykardie	0,8	1,0
Palpitationen	0,8	0,9
Hypotonie	0,8	0,8
Halluzinationen	0,8	0,7
Sinnestäuschungen	0,7	0,5
Kopfschmerzen	0,5	0,3
Angst/Ängste/Panik/Albträume	0,5	0,4
Hypertonie	0,4	0,2
Dysarthrie	0,3	0,6
Wahnvorstellungen	0,3	0,5
Allergien/Hautausschlag	0,3	0,1
Schwitzen	0,3	0,3
Dissoziation	0,2	0,3
Unruhe	0,2	0,2
Benommenheit	0,2	0,3
Suizidgedanken	0,1	0,3
Aggressivität	0,1	0,1
Husten	0,1	0,3
Konzentrationsstörungen	0,1	0,1
Sucht/Abhängigkeit/Missbrauch/Abusus	0,0	0,1
Wesensveränderung	0,0	0,1

Mehrfachnennungen sind möglich

Bei den 3499 Therapieabbrechern war Müdigkeit (20 %) die am häufigsten mitgeteilte Nebenwirkung, gefolgt von Schwindel (18 %), Schläfrigkeit (10 %), Aufmerksamkeitsstörungen (8 %), Gleichgewichtsstörungen (7 %), Mundtrockenheit (6 %), Gedächtnisstörungen (5 %), Desorientierung (4 %), Lethargie (3 %), Depression (2,6 %), Erbrechen (2 %), Tachykardie (2 %), Halluzinationen (1,7 %), Sinnestäuschungen (1,3 %), Wahnvorstellungen (0,9 %), Dissoziation (0,5 %) und Suizidgedanken (0,4 %).

### Missbrauch, Beigebrauch, Kombination mit Opioiden

Missbrauch von Cannabisarzneimitteln und Beigebrauch von illegalem Cannabis wurden in der Begleiterhebung in 27 Fällen im Freitext berichtet, auch als Grund für einen Therapieabbruch.

Bei Verschreibung von Cannabisblüten zur Behandlung von Schmerzen (als Hauptdiagnose) wurde seltener gleichzeitig mit Opioiden therapiert als bei der Verschreibung anderer Cannabisarzneimittel (24 % vs. 37,6 %).

### Anwendung, Dosierung von Cannabisblüten

Cannabisblütensorten mit einem THC-Gehalt von > 20 % wurden bevorzugt angewendet (1027 [77 %] von 1343 auswertbaren Fällen). In 207 Fällen wurden bei der ersten Verordnung mehrere (bis zu 4) Blütensorten verschrieben. In 782 Fällen (43 %) blieben im Verlauf der Therapie die Dosierung, die Sorte und die Art der Anwendung unverändert. In 475 Fällen (26 %) wurde die Dosierung erhöht, in 61 Fällen (3 %) vermindert, in 667 Fällen (37 %) wurde die Sorte und in 106 Fällen (6 %) die Art der Anwendung umgestellt. Bei Erstverordnung der Blüten lag die Dosis durchschnittlich bei einem Gramm pro Tag (Median 0,81 g bei 1206 auswertbaren Fällen), die Anwendung erfolgte in 1259 Fällen über die Atemwege mit Verdampfung/Inhalation/Vaporizer, in 75 Fällen als Tee. In 25 Fällen wurde Rauchen als Anwendungsart angegeben.

### Vorangegangene Behandlungen mit Cannabisarzneimitteln

612 Patienten (6,1 % aller Fälle und 15 % der Patienten, welche zuletzt mit Cannabisblüten therapiert wurden) waren bereits vor dem am 10.03.2017 in Kraft getretenen Gesetz zur Änderung betäubungsmittelrechtlicher und anderer Vorschriften im Besitz einer Ausnahmeerlaubnis nach § 3 Absatz 2 Betäubungsmittelgesetz (BtMG) zum Erwerb von Cannabis zur Anwendung im Rahmen einer medizinisch betreuten und begleiteten Selbsttherapie und hatten Zugang zur Therapie mit Cannabisblüten oder -extrakt.

In 1312 Fällen (13,1 %) wurde angegeben, dass bereits vor der Verschreibung eines Cannabisarzneimittels auf Grundlage der Genehmigung durch die Krankenkasse eine Therapie mit einem anderen Cannabisarzneimittel durchgeführt wurde, zumeist mit Sativex® (548 Fälle) oder Dronabinol (517 Fälle).

### Therapieerfolg

Bei den 7312 Fällen mit Schmerz als primär behandeltem Symptom wurde der Therapieerfolg in 34 % als *deutlich verbessert* beurteilt, in 36 % als *moderat verbessert* und in 28 % als *unverändert*.

Bei 113 Patienten mit multipler Sklerose als erster Hauptdiagnose, bei welchen gleichzeitig als primär therapierte Symptomatik Spastik angegeben wurde, wurde der Therapieerfolg von Dronabinol bei 41 % als *deutlich verbessert*, bei 43 % als *moderat verbessert*, bei 15 % als *unverändert* und bei einem Patienten als *deutlich verschlechtert* beurteilt, wobei in dieser Patientengruppe Unterschiede zwischen Männern und Frauen bezüglich des Therapieerfolgs bestanden. Bei Männern (34 Fälle) wurde der Therapieerfolg in 29 % der Fälle mit *deutlich verbessert*, in 50 % mit *moderat verbessert*, bei 21 % als *unverändert* angegeben. Die Spastik bei Frauen (78 Fälle) zeigte sich in 46 % der Fälle *deutlich verbessert*, in 40 % *moderat verbessert* und in 13 % *unverändert*.

Die Datensätze zur Verschreibung von Dronabinol zur Appetitförderung, bei denen nicht Anorexie/Wasting, sondern *Sonstiges* als Diagnose ausgewählt wurde, wurden zusammengefasst ausgewertet. Die Zuordnung der Diagnosen ergab sich aus den Freitextangaben, wie z. B. Inappetenz, Appetitsteigerung, Appetitlosigkeit, Appetitverlust. Bei einer Fallzahl von 125 wurde in 22 % der Fälle der Therapieerfolg als *deutlich verbessert*, bei 42 % als *moderat verbessert* und bei 36 % als *unverändert* eingeschätzt. Der Anteil der deutlichen Verbesserung war bei beiden Geschlechtern gleich, bei männlichem Geschlecht (*n* = 46) betrug der Anteil der moderaten Verbesserung 52 %, unverändert blieb die Symptomatik bei 26 %. Bei weiblichem Geschlecht (*n* = 79) betrug der Anteil der moderaten Verbesserung 37 %, unverändert blieb die Symptomatik bei 42 %.

## Diskussion

Die Begleiterhebung erfasst nicht alle in Deutschland mit Cannabisarzneimitteln behandelten Patientinnen/Patienten. Die Daten von Selbstzahlern, Privatversicherten, stationär und im Rahmen von klinischen Studien behandelten Personen werden nicht übermittelt. Fälle, in denen die Fertigarzneimittel Sativex® und Canemes® entsprechend den zugelassenen Anwendungsgebieten verordnet werden, werden in der Begleiterhebung nicht erfasst. Es besteht ärztlicherseits eine Verpflichtung zur Teilnahme an der Begleiterhebung, der offensichtlich nicht in jedem Einzelfall nachgekommen wird. Mehr als 3 Jahre nach Beginn der Begleiterhebung sind ca. 10.000 Datensätze eingegangen. Bis zum Ende des Jahres 2019 war in nahezu 18.000 Fällen eine Aufklärung der Patientinnen und Patienten bezüglich der verpflichtenden Teilnahme an der Begleiterhebung erfolgt und in 9000 Fällen ist eine Datenübermittlung abgerechnet worden. In mehr als 27.000 Fällen wurde ärztlicherseits eine Gebühr zur Unterstützung der Antragsteller zur Cannabistherapie mit einer gesetzlichen Krankenkasse abgerechnet [31]. In Verbindung mit Informationen der Krankenkassen [7] ist davon auszugehen, dass bis Mai 2020 mehr Rückmeldungen in der Begleiterhebung hätten erfolgen müssen.

Während in der Begleiterhebung 49 % der Eingaben von Anästhesistinnen/Anästhesisten stammen, sind diese nach Angaben der Barmer-Krankenkasse für weniger als ein Viertel der Verordnungen verantwortlich. Hausärztinnen/Hausärzte (Allgemeinmedizin, praktische Ärzte, innere Medizin) hingegen sind für mehr als 40 % der Verordnungen verantwortlich, in der Begleiterhebung jedoch nur zu 27 % vertreten [30]. Da Hausärztinnen/Hausärzte mehr als 50 % der Cannabisblüten verordnen, dürfte die Meldelücke überwiegend die Verordnung von Cannabisblüten betreffen, wie sich auch aus dem Vergleich der Verordnungszahlen in der GKV-Arzneimittel-Schnellinformation (GAMSI; [7]) mit den Anteilen der Meldungen zu den jeweiligen Cannabisarzneimitteln in der Begleiterhebung ableiten lässt. Für die Fertigarzneimittel Sativex® und Canemes® ist zu berücksichtigen, dass in den Daten der Krankenkassen, anders als in der Begleiterhebung, auch die In-label-Verordnungen, d. h. entsprechend den zugelassenen Anwendungsgebieten, enthalten sind.

**Figure 4:**
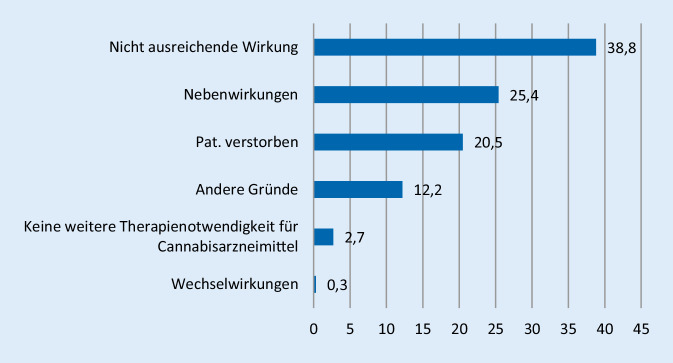

Die Ergebnisse der ersten Zwischenauswertung aus Februar 2019 wurden bezüglich der Häufigkeitsverteilung von Indikationen, die eine Behandlung mit Cannabisarzneimitteln begründen, bestätigt [1]. In nahezu drei Vierteln der Fälle werden Schmerzen als primär therapierte Symptomatik genannt. Die Diagnose Aufmerksamkeitsdefizit-Hyperaktivitätsstörung (ADHS), die vor dem Jahr 2017 bei den erteilten Ausnahmeerlaubnissen zum Erwerb von Cannabis zu medizinischen Zwecken in 14 % der Fälle als primär behandelte Diagnose genannt wurde, hat lediglich einen Anteil von gut einem Prozent der in der Begleiterhebung erfassten Fälle. Einfluss mag darauf auch die Empfehlung in der 2017 veröffentlichten Leitlinie der Arbeitsgemeinschaft der Wissenschaftlichen Medizinischen Fachgesellschaften (AWM;) [8]) haben, Cannabis für die Behandlung der ADHS nicht einzusetzen. Häufigster Grund für einen Therapieabbruch war unverändert bei einem Drittel der Fälle die nicht ausreichende Wirkung (Abb. 4).

Die Behandlung mit Cannabisblüten erfolgt wesentlich häufiger bei Männern als bei Frauen. Diese Patienten mit Cannabisblütenverordnung sind zudem im Durchschnitt deutlich jünger als jene, die mit den anderen Cannabisarzneimitteln therapiert werden. Häufiger als im Durchschnitt aller Behandelten sind auch Vorerfahrungen mit einer Cannabistherapie. Diese Patientengruppe berichtete weniger häufig über Nebenwirkungen, wurde länger behandelt und brach seltener die Therapie ab. Dies spricht im Zusammenhang mit den über die Ausnahmeerlaubnisse objektivierbaren häufigeren Vorerfahrungen für einen verhältnismäßig hohen Anteil an Behandelten mit vorheriger Gewöhnung. Der geringere Anteil der Tumor- bzw. palliativ behandelten Fälle ist einer der Gründe für die vergleichsweise längere Behandlungsdauer in dieser Patientengruppe. Der höhere Anteil anderer Symptomatiken (als Schmerz, Spastik, Anorexie/Wasting) als Therapiegrund tritt gleichzeitig mit einem höheren Anteil Verschreibender aus dem Fachgebiet Allgemeinmedizin auf. Ärztinnen/Ärzte mit den Zusatzbezeichnungen *Schmerz- und Palliativmedizin* verordnen seltener Cannabisblüten, auch wenn in dieser Patientengruppe Schmerz ebenfalls die häufigste primär behandelte Symptomatik ist (Abb. 2).

Die Nebenwirkungshäufigkeiten aus den ersten Zwischenauswertungen [1, 3] wurden bestätigt, wobei die Zahl von mehr als 10.000 Fällen nun auch Aussagen zu nicht häufigen Nebenwirkungen erlaubt. Am häufigsten traten, wie in der ersten Zwischenauswertung, psychiatrische Symptome und neurologische Störungen als Nebenwirkungen auf. Auswirkungen auf die Kognition werden in der Begleiterhebung als Gedächtnisstörungen (3 % der Fälle) und Aufmerksamkeitsstörungen (5 % der Fälle) berichtet. Patientinnen/Patienten, die keine Nebenwirkungen angaben, sind bei der Behandlung mit Dronabinol (57 %) und Cannabisblüten (62 %) deutlich häufiger als bei der Behandlung mit Sativex® (38 %). Dies ist u. a. bedingt durch Nebenwirkungen im Mund-Rachen-Raum. Bei Sativex® wurde ein deutlich höherer Anteil anderer Nebenwirkungen (16 % vs. jeweils 7 % bei Dronabinol und Cannabisblüten) angegeben, die insbesondere als lokale Unverträglichkeit des Mundsprays beschrieben wurden. Darüber hinaus wurde häufiger als bei den anderen Cannabisarzneimitteln über Übelkeit und Erbrechen, verursacht durch unangenehmen Geschmack, berichtet. Die aus der Literatur bekannten kardiovaskulären Risiken [9] werden durch die in der Begleiterhebung im Freitext gemeldeten 9 kardialen Fälle (darunter ein Herzinfarkt, 2 Fälle von Verschlechterung einer Herzinsuffizienz und 2 Fälle von Bradykardie) bestätigt, zusätzlich wurden 90 Fälle mit Tachykardie und 87 Fälle mit Palpitationen (Wahrnehmung eines ungewöhnlichen Herzschlags) berichtet.

Die Nebenwirkungsmeldungen in der Begleiterhebung können nicht die Meldung unerwünschter Arzneimittelwirkungen (UAW) im Rahmen des Spontanmeldesystems ersetzen, da eine Überprüfung der Einzelfälle aufgrund der Anonymisierung nicht erfolgen kann. Die Nebenwirkungen entsprechen den Angaben in den Fachinformationen zu Sativex®, Canemes® und Marinol® [4–6]. Zu den weiteren cannabisbasierten Arzneimitteln und zu Cannabisblüten fehlen Fachinformationen, Zulassung und Zulassungsstudien. In der Begleiterhebung wurde die Nebenwirkung Depression (1,3 %) häufig (1–10 Behandelte von 100) und Dissoziation (0,2 %), Suizidgedanken (0,2 %), Halluzination (0,8 %) und Wahnvorstellungen (0,4 %) jeweils gelegentlich (1–10 Behandelte von 10.000) gemeldet (Abb. 3). Diese Nebenwirkungen sind definitionsgemäß *potenziell schwerwiegend* und somit von hoher Relevanz für eine sichere Anwendung von Cannabisarzneimitteln, ebenso wie der in mehr als 10 % der Fälle auftretende Schwindel.

In einer Analyse italienischer Pharmakovigilanzdaten zu Cannabisblüten wurden von 53 Nebenwirkungsmeldungen 12 Nebenwirkungen als schwerwiegend bewertet. Am häufigsten wurden psychiatrische und neurologische Störungen gemeldet (26 % bzw. 22 %; [10]). Dies entspricht den Ergebnissen der Begleiterhebung, wonach psychiatrische und neurologische Nebenwirkungen etwa doppelt so häufig angegeben werden wie Nebenwirkungen anderer Organsysteme.

Dass die Nebenwirkungsprofile von Dronabinol (als reines THC) und den weiteren, auch CBD-haltigen Cannabisarzneimitteln sich nicht wesentlich unterscheiden (Tab. 2), spricht gegen die aus Tierversuchen abgeleitete Annahme, dass THC durch die Beimischung von CBD verträglicher wird [11].

In der Subgruppenanalyse von Behandlungen einer Spastik bei multipler Sklerose mit Dronabinol zeigte sich in der Begleiterhebung bei Frauen ein etwas besserer Therapieerfolg als bei Männern. Dieser Unterschied wäre, auch unter Berücksichtigung der zu dem ebenfalls THC-haltigen Sativex® berichteten Effektmodifikation durch das Subgruppenmerkmal Geschlecht (vgl. [12]), ggf. in einer klinischen Studie weiter zu untersuchen.

Die 5‑stufige Beurteilung des Therapieerfolgs und der Änderung der Lebensqualität in der Begleiterhebung durch den behandelnden Arzt erlaubt keine klare Unterscheidung zwischen Arzneimittelwirkung, Suchtstoff- und Placeboeffekten. Allerdings ist bei ausreichender Fallzahl, unter Berücksichtigung der Abbruchrate und der Nebenwirkungsprofile, die Beurteilung der Effizienz einer Behandlung grundsätzlich möglich. Die Begleiterhebung kann jedoch klinische Studien nicht ersetzen.

Durch kürzlich veröffentlichte Studienergebnisse werden die Indikationen der Fertigarzneimittel Sativex® und Canemes® als Zweitlinientherapie bei multipler Sklerose [13] bzw. bei chemotherapiebedingter Emesis und Nausea [14] bestätigt. Die Ergebnisse der Begleiterhebung sind mit diesen Studienergebnissen nicht vergleichbar, da die der Zulassung entsprechende Anwendung von Sativex® nicht erfasst wird bzw. hinsichtlich Emesis und Nausea in der Begleiterhebung nicht unterschieden wird, ob die Symptomatik bei vorliegender Krebserkrankung chemotherapiebedingt ist.

Positive Therapieeffekte von Nabilon bei Parkinson, wie sie in einer kürzlich veröffentlichten Studie zu Patienten mit nichtmotorischen Störungen bei Parkinson beschrieben wurden [15], lassen sich mit den vorliegenden Daten nicht belegen, da in der Begleiterhebung bisher zu wenige Fälle mit Parkinsonsyndrom (in 28 Fällen als erste Hauptdiagnose, davon keiner mit Nabilon behandelt) vorliegen.

Ein Vergleich der Wirksamkeit der Cannabisarzneimittel sollte wegen der deutlichen demografischen Unterschiede als altersstandardisierte Untergruppenanalyse erfolgen. Dafür waren die vorliegenden Fallzahlen zu Cannabisblüten, Sativex®, Nabilon und Cannabisextrakten allerdings noch zu gering. Wechselwirkungen wurden in der Begleiterhebung nur selten als Grund für einen Therapieabbruch erfasst (11 Fälle), wobei sowohl für THC als auch für CBD Arzneimittelinteraktionen bekannt sind [16].

Die fehlenden wissenschaftlichen Daten zu Wirksamkeit und Sicherheit von Cannabisarzneimitteln [17–29] in Verbindung mit einer vergleichsweise hohen Nebenwirkungsrate und dem nicht seltenen Auftreten potenziell schwerwiegender Nebenwirkungen lassen eine Anwendung dieser Arzneimittel nur im Ausnahmefall zu. Insofern muss die gesetzliche Anforderung nochmals bekräftigt werden, Cannabisarzneimittel nur anzuwenden, wenn andere Arzneimittel nicht wirksam sind oder aus anderen Gründen nicht angewendet werden können.
